# Flexible sigmoidoscopy in colorectal cancer screening: implications of different colonoscopy referral strategies

**DOI:** 10.1007/s10654-018-0404-x

**Published:** 2018-05-12

**Authors:** Tobias Niedermaier, Korbinian Weigl, Michael Hoffmeister, Hermann Brenner

**Affiliations:** 1 Division of Clinical Epidemiology and Aging Research, German Cancer Research Center (DKFZ), Im Neuenheimer Feld 581, 69120 Heidelberg, Germany; 2 Medical Faculty Heidelberg, University of Heidelberg, Heidelberg, Germany; 3 Division of Preventive Oncology, German Cancer Research Center (DKFZ) and National Center for Tumor Diseases (NCT), Heidelberg, Germany; 4 German Cancer Consortium (DKTK), German Cancer Research Center (DKFZ), Heidelberg, Germany

**Keywords:** Colorectal neoplasia, Detection, Screening, Colonoscopy referral

## Abstract

Flexible sigmoidoscopy (FS) screening reduces colorectal cancer incidence and mortality. Its potential to detect proximal neoplasms depends on colonoscopy referral. We estimated diagnostic performance of sigmoidoscopy using 12 different referral criteria in detecting colorectal cancer and advanced adenomas. Colonoscopy results from 14,947 participants of screening colonoscopy in Germany were used to derive sensitivity of sigmoidoscopy for colorectal cancer, advanced adenomas (AAs), and any advanced neoplasms in the proximal colon. It was assumed that FS detects the same neoplasms as colonoscopy within its reach and that distal neoplasms would be followed by colonoscopy. In addition, numbers of colonoscopies needed (NCN) to detect one proximal advanced neoplasm were calculated. The most advanced findings during colonoscopy were colorectal cancer in 213 subjects (1.4%), AA in 1539 subjects (10.2%) and non-advanced adenomas in 2988 subjects (19.8%). Without colonoscopy referral, overall sensitivities for any colorectal cancer, advanced adenoma and any advanced neoplasm (proximal or distal) would be 79, 65 and 66%, respectively. These sensitivities could be increased to up to 86, 83 and 84% by the referral strategies investigated. Compared to referral due to advanced adenomas, referral due to non-advanced adenomas would substantially increase the NCN at a modest gain in sensitivity. Sensitivities were higher and NCNs were lower in men than in women for every strategy. In conclusion, colonoscopy referral can substantially increase sensitivity of sigmoidoscopy-based screening, but the gain by referral due to non-advanced adenomas substantially increases NCN compared to referral due to advanced neoplasms only. Major sex differences may call for sex-specific referral strategies.

**Electronic supplementary material:**

The online version of this article (10.1007/s10654-018-0404-x) contains supplementary material, which is available to authorized users.

## Introduction

Colorectal cancer (CRC) is a common, yet largely preventable disease. Although colonoscopy is the gold standard in CRC and advanced adenoma detection, disadvantages such as higher costs, discomfort and complication rates as well as lower adherence limit its use as primary screening method. Flexible sigmoidoscopy (FS) reliably detects colonic neoplasms in the distal colon and rectum, which is where the majority of all colorectal neoplasms occur [1]. Proximal neoplasms can be detected by FS only indirectly if detection of distal lesions is followed by colonoscopy, as recommended in CRC screening guidelines [2–4]. Several randomized clinical trials (RCTs) demonstrated that FS screening reduces CRC incidence and mortality [5–8]. FS screening is offered in Italy, UK and in the USA [9].

The RCTs on effectiveness of FS screening differed in their colonoscopy referral criteria [5–8]. While the UK Flexible Sigmoidoscopy Trial [10] imposed the highest thresholds for colonoscopy referral, less restrictive criteria were imposed in the SCORE trial (Italy) [5], the PLCO trial (USA) [6] and the NORCCAP trial (Norway) [11]. Accordingly, colonoscopy referral rates and detection rates of proximal colonic neoplasms differed between these trials [12].

More restrictive referral strategies imply lower detection rates than less restrictive criteria, but also lower numbers of colonoscopies needed (NCN) to detect one relevant proximal finding [12]. Comparative evaluations of various potential colonoscopy referral strategies within the same study population are sparse [13–15]. Differential performance of the same strategies in men and women can be expected due to the typically higher prevalence of colorectal neoplasia among men. In Germany, FS is not routinely conducted for primary CRC screening, despite proven effectiveness and lower costs and effort compared to colonoscopy screening. We used colonoscopy findings in a large German CRC screening population to compare the expected impact on diagnostic performance (sensitivities and NCN) of 12 referral strategies following screening FS in men and women.

## Materials and methods

### Study design and study population

We used data from the ongoing KolosSal study, which has been described elsewhere [16, 17]. In this statewide cohort study, initiated in 2005 in Saarland, a small state (1 million inhabitants) in southwestern Germany, CRC incidence and mortality are monitored among participants of screening colonoscopy. For our analysis, we used baseline data from participants recruited in 33 gastroenterology practices in Saarland from January 2, 2006, through October 31, 2012.

In the German screening colonoscopy program, subjects aged ≥ 55 years (no upper age limit) are eligible for screening colonoscopy, with the option of a second screening colonoscopy ≥ 10 years later. Almost all screening colonoscopies are conducted in practices of gastroenterology or internal medicine. To become eligible, endoscopists must have conducted ≥ 200 colonoscopies and ≥ 50 polypectomies under supervision in the preceding two calendar years. To maintain eligibility, endoscopists must conduct ≥ 200 colonoscopies per year and ≥ 10 polypectomies per year. Histopathologic examination of removed polyps is performed decentrally; endoscopists send polyps to a certified pathological laboratory of their choice.

Nearly all practices conducting screening colonoscopies in Saarland agreed to recruit patients for the study cohort. Eligible patients had to be residents of Saarland undergoing screening colonoscopy in a participating practice. 18,997 subjects were recruited between January 2, 2006, and October 31, 2012. The study was approved by ethics committees of the University of Heidelberg and of the Medical Association of Saarland. Each participant provided written informed consent.

**Fig. 1:**
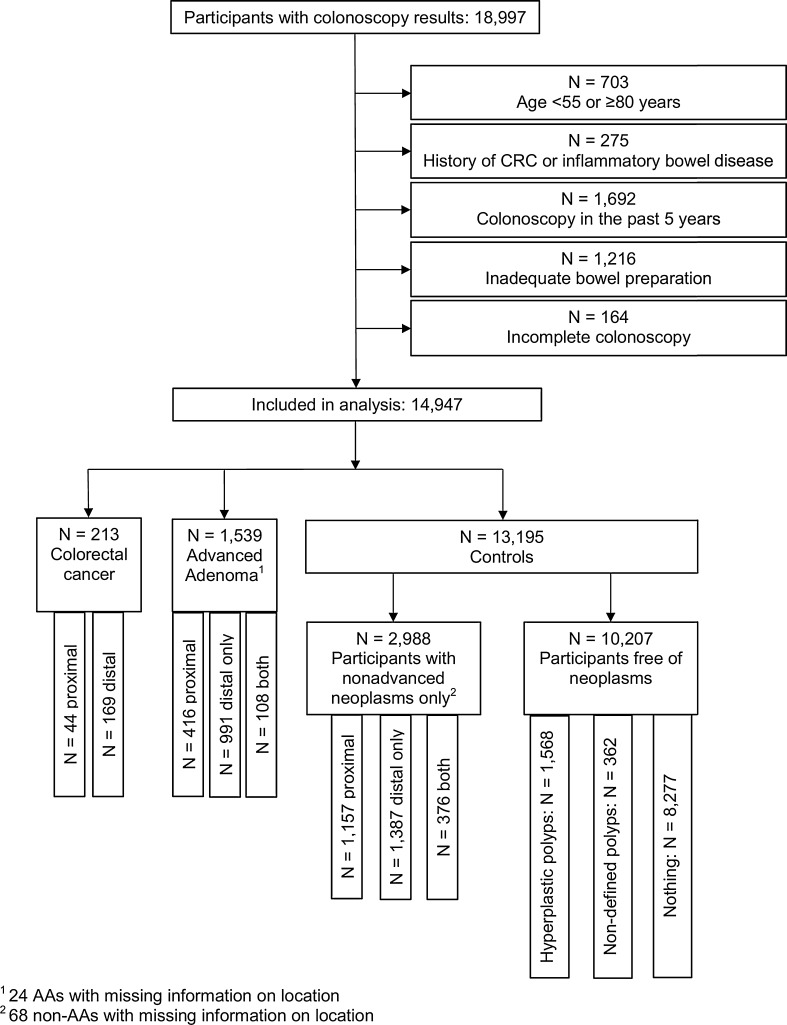
Flow diagram of the participants in the KolosSal study included in this analysis

Representativeness of our results for an average-risk screening population was ensured by excluding participants matching any of the following criteria (Fig. 1): < 55 or ≥ 80 years of age (N = 703), history of CRC or inflammatory bowel disease (N = 275); colonoscopy in the preceding 5 years (N = 1692). To minimize the number of screening colonoscopies with missed neoplasms, subjects with inadequate bowel preparation before colonoscopy (N = 1216) or incomplete colonoscopy (coecum not reached; N = 164) were excluded. Thus, 14,947 participants were retained for the analysis. Approximately one fourth of them (23.5%, N = 3499) had a previous colonoscopy more than 5 years ago.

### Data collection

Participants were recruited in the practices prior to screening colonoscopy, typically at a preparatory visit. They provided basic information on CRC risk and preventive factors in a standardized questionnaire and agreed that copies of colonoscopy and histology reports were forwarded by the physician for this study. Those reports were reviewed by trained investigators who were blinded with respect to questionnaire data. Participants were classified into the following categories according to the most advanced finding at colonoscopy: CRC, advanced adenoma (AA), nonadvanced adenoma, other. Adenomas with at least one of the following features were defined as AAs: size ≥ 1 cm, tubulo-villous or villous components, high-grade dysplasia.

### Statistical analyses

Sensitivity of FS was derived from colonoscopy results. FS was assumed to detect the same neoplasms as colonoscopy within its reach. Proximal and distal location of neoplasms were defined according to the assumed reach of FS. In the main analyses, colonoscopic findings were defined as proximal (FS-unreachable) when located proximal to the descending colon and distal otherwise. In sensitivity analyses, findings located proximal to the sigmoid colon were defined as proximal, otherwise as distal. In addition, expected detection of proximal neoplasms due to different referral to colonoscopy after detection of distal neoplasms was examined.

We investigated the referral criteria of the FS screening trials (UK, SCORE, NORCCAP, US/PLCO, see Table 1) and further recommended or conceivable referral strategies. These strategies used the following referral criteria (based on distal findings) and were sorted according to the number of colonoscopy referrals: ≥ 2 neoplasms, thereof ≥ 1 advanced neoplasm (AN, defined as CRC or AA); ≥ 2 neoplasms; ≥ 1 histology-defined AN (high-grade dysplasia, tubulo-villous components or both); AN ≥ 1 cm; any AN; any neoplasm; any neoplasm or hyperplastic polyp (defined as such by the pathological labs, excluding adenomatous polyps, serrated polyps, pseudopolyps and other findings).

**Table 1:** Investigated colonoscopy referral criteria according to findings at flexible sigmoidoscopy

Referral criteria	Details	Ref.
Trials		
UK FS screening trial	CRC, one distal polyp or adenoma > 1 cm, (tubulo-)villous histology, HGD, ≥ 3 adenomas or ≥ 20 hyperplastic polyps above the rectum	[10]
SCORE	Distal polyp(s) > 5 mm, (tubulo-)villous histology, HGD, ≥ 3 adenomas or CRC	[5]
NORCCAP	CRC, one distal polyp ≥ 1 cm or any adenoma	[11]
*US PLCO trial*	*Score *≥* 4 [age (50*–*54: 0, 55*–*59: 1, 60*–*64: 2, 65*–*70: 3) *+* gender (female: 0, male: 1) *+* most advanced distal finding (no polyps: 0, hyperplasia: 1, tubular adenoma *<* 10* *mm: 2, advanced lesion (tubular adenoma *≥* 10* *mm, villous histology, HGD, CRC: 3)]*	[6]
Other		
≥* 2 neoplasms, *≥* 1 AN*	*At least two distal adenomas, thereof at least one advanced adenoma, or CRC*	
≥* 2 neoplasms*	*At least two distal adenomas or CRC*	
*Histology-defined AN*	*Distal (tubulo-)villous adenoma or HGD or CRC*	
*AN *≥* 1* *cm*	*Distal large (*≥* 1* *cm) adenoma or CRC*	
Any AN	Any distal advanced adenoma or CRC	
Any neoplasm	Any distal adenoma or CRC; Recommended by several guidelines^a^	[2–4]
Any neoplasm or HPP	Any distal adenoma or hyperplastic polyp or CRC	

Strategies in *italic* would not automatically refer subjects with any distal AN to colonoscopy

*AN* advanced neoplasia, *CRC* colorectal cancer, *HGD* high-grade dysplasia, *HPP* hyperplastic polyp, *Ref.*, reference, *NORCCAP* Norwegian Colorectal Cancer Prevention, *PLCO* Prostate, Lung, Colorectal, and Ovarian Cancer, *SCORE* Screening for COlon Rectum

^a^Referral criteria recommended in guidelines by the American College of Gastroenterology, the American Cancer Society, Group Health Cooperative, American Cancer Society, US Multi-Society Task Force on Colorectal Cancer, and the American College of Radiology [2–4]

#### Outcomes

Overall sensitivities for detecting (proximal or distal) CRC, AAs or any AN were investigated as outcomes for the aforementioned referral criteria. Sensitivities were calculated as the number of subjects with AN correctly identified by FS itself or colonoscopy referral divided by the total number of subjects with AN detected during colonoscopy.

In addition, we calculated the NCN per “FS-unreachable” (proximal) outcome (CRC, AA, any AN). This number equals the number of participants with a neoplasm in the distal colon or rectum that would lead to follow-up colonoscopy (which differs between the investigated strategies), divided by the number of participants in whom a proximal AN would be detected by follow-up colonoscopy. To investigate the burden and benefit of relaxing referral criteria, we calculated the number of additional colonoscopies needed to detect one additional proximal AN when comparing more extensive to the most restrictive examined referral strategy (“incremental NCN”). Finally, negative predictive values were calculated for all outcomes and referral strategies, i.e., the probability of a subject that would not be referred to colonoscopy having no proximal AN.

All outcome measures (sensitivities, NCN) were calculated stratified by gender and for the entire study population.

Statistical analyses were performed in R [18] version 3.2.5. For sensitivities, 95% Clopper-Pearson (binomial) confidence intervals (CIs) were calculated using the R package “binom” [19] version 1.1-1.

## Results

### Study population

The study population comprised 14,947 subjects. 49.0% were male. Mean age was 63.2 years, 213 subjects (1.4%) had CRC, 1539 (10.3%) had AAs and 2988 (20.0%) had non-advanced adenomas as the most advanced findings at screening colonoscopy (Table 2). Prevalences of CRC and AA were approximately twice as high in men as in women.

**Table 2:** Characteristics of the KolosSal study population

Characteristic	Total, N = 14,947		Men, N = 7323		Women, N = 7624	
	N	%	N	%	N	%
Age (years)						
55–59	5672	37.9	2680	36.6	2992	39.2
60–64	3263	21.8	1608	22.0	1655	21.7
65–69	3049	20.4	1527	20.9	1522	20.0
70–74	2077	13.9	1045	14.3	1032	13.5
75–79	886	5.9	463	6.3	423	5.5
Most advanced finding at screening colonoscopy						
Colorectal cancer	213	1.4	140	1.9	73	1.0
Advanced adenoma	1539^a^	10.3	985	13.5	554	7.3
Non-advanced adenoma	2988	20.0	1721	23.5	1267	16.6
Hyperplastic polyps	1568	10.5	796	10.9	772	10.1
Other/unspecified polyps	362	2.4	192	2.6	170	2.2
No finding	8277	55.4	3489	47.6	4788	62.8

^a^Thereof 24 AA cases with missing information on location, leaving 1515 AA cases for analyses

### Diagnostic performance of FS

Expected diagnostic performance of FS, assuming different colonoscopy referral strategies following FS reaching the descending colon, is summarized in Table 3. Consistently lower sensitivities were observed in women than in men. Sensitivity for CRC without any colonoscopy referral was estimated as 84% (95% CI 77–90%) in men and 70% (95% CI 58–80%) in women. 67% of AAs in men and 63% of AAs in women were within the reach of FS. In men and women, a steady increase in sensitivities was observed when assuming colonoscopy referral and applying gradually less restrictive referral criteria. Up to 85% of AN would have been detected in men and up to 76% in women, assuming referral due to any neoplasm or hyperplastic polyp. All sensitivities were lower by approximately 5 percentage points when assuming that FS does not reach and visualize the descending colon (Supplementary Table 1).

**Table 3:** Expected overall sensitivities in % (95% CIs) of flexible sigmoidoscopy (FS) based screening with different colonoscopy referral strategies in the male and female KolosSal study population, sorted by the number of colonoscopies conducted

Sex	Colonoscopy referral criterion after FS screening	Number of colonoscopies	Most advanced finding at colonoscopy					
			CRC (N = 140/73)		AA (N = 971/544)		Any AN (N = 1111/617)	
			N detected	Sensitivity [%] (95% CI)	N detected	Sensitivity [%] (95% CI)	N detected^b^	Sensitivity [%] (95% CI)
Men	*No referral*	*0*	*118*	*84 (77*–*90)*	*646*	*67 (63*–*69)*	*752*	*68 (65*–*70)*
	UK FS screening trial	965	126	90 (84–94)	740	76 (73–79)	866	78 (75–80)
	SCORE	1146	126	90 (84–94)	751	77 (75–80)	877	79 (76–81)
	NORCCAP	2004	127	91 (85–95)	787	81 (78–83)	914	82 (80–84)
	*US (PLCO)*	*4167*	*135*	*96 (92*–*99)*	*887*	*91 (89*–*93)*	*1022*	*92 (90*–*94)*
	≥*2 neoplasms, *≥* 1 AN*	*395*	*122*	*87 (80*–*92)*	*686*	*71 (68*–*73)*	*808*	*73 (70*–*75)*
	≥*2 neoplasms*	*630*	*123*	*88 (81*–*93)*	*703*	*72 (69*–*75)*	*826*	*74 (72*–*77)*
	*Histology*-*defined AN*^*a*^	*617*	*124*	*89 (82*–*93)*	*703*	*72 (69*–*75)*	*827*	*74 (72*–*77)*
	*AN *>* 1* *cm*	*639*	*122*	*87 (80*–*92)*	*706*	*73 (70*–*75)*	*828*	*75 (72*–*77)*
	Any AN	854	125	89 (83–94)	729	75 (72–78)	854	77 (74–79)
	Any neoplasm	1941	127	91 (85–95)	781	80 (78–83)	908	82 (79–84)
	Any neoplasm or HPP	2737	127	91 (85–95)	816	84 (82–86)	943	85 (83–87)
Women	*No referral*	*0*	*51*	*70 (58*–*80)*	*345*	*63 (59*–*67)*	*395*	*64 (60*–*68)*
	UK FS screening trial	456	52	71 (59–81)	372	68 (64–72)	424	69 (65–72)
	SCORE	586	52	71 (59–81)	377	69 (65–73)	429	70 (66–73)
	*US (PLCO)*	*1062*	*57*	*78 (67*–*87)*	*388*	*71 (67*–*75)*	*445*	*72 (68*–*76)*
	NORCCAP	1216	57	78 (67–87)	396	73 (69–76)	453	73 (70–77)
	≥*2 neoplasms, *≥* 1 AN*	*152*	*52*	*71 (59*–*81)*	*354*	*65 (61*–*69)*	*406*	*66 (62*–*70)*
	≥*2 neoplasms*	*263*	*53*	*73 (61*–*82)*	*358*	*66 (62*–*70)*	*411*	*67 (63*–*70)*
	*Histology*-*defined AN*^*a*^	*307*	*52*	*71 (59*–*81)*	*363*	*67 (63*–*71)*	*415*	*67 (63*–*71)*
	*AN *>* 1* *cm*	*314*	*52*	*71 (59*–*81)*	*359*	*66 (62*–*70)*	*411*	*67 (63*–*70)*
	Any AN	422	52	71 (59–81)	370	68 (64–72)	422	68 (65–72)
	Any neoplasm	1186	57	78 (67–87)	394	72 (68–76)	451	73 (69–77)
	Any neoplasm or HPP	1874	57	78 (67–87)	412	76 (72–79)	469	76 (72–79)
Both sexes	*No referral*	*0*	*169*	*79 (73*–*85)*	*991*	*65 (63*–*68)*	*1147*	*66 (64*–*69)*
	UK FS screening trial	1421	178	84 (78–88)	1112	73 (71–76)	1290	75 (73–77)
	SCORE	1732	178	84 (78–88)	1128	74 (72–77)	1306	76 (73–78)
	NORCCAP	3220	184	86 (81–91)	1183	78 (76–80)	1367	79 (77–81)
	*US (PLCO)*	*5229*	*192*	*90 (85*–*94)*	*1275*	*84 (82*–*86)*	*1467*	*85 (83*–*87)*
	≥*2 neoplasms, *≥* 1 AN*	*547*	*174*	*82 (76*–*87)*	*1040*	*69 (66*–*71)*	*1214*	*70 (68*–*72)*
	≥*2 neoplasms*	*893*	*176*	*83 (77*–*87)*	*1061*	*70 (68*–*72)*	*1237*	*72 (69*–*74)*
	*Histology*-*defined AN*^*a*^	*924*	*176*	*83 (77*–*87)*	*1066*	*70 (68*–*73)*	*1242*	*72 (70*–*74)*
	*AN *>* 1* *cm*	*953*	*174*	*82 (76*–*87)*	*1065*	*70 (68*–*73)*	*1239*	*72 (70*–*74)*
	Any AN	1276	177	83 (77–88)	1099	73 (70–75)	1276	74 (72–76)
	Any neoplasm	3127	184	86 (81–91)	1175	78 (75–80)	1359	79 (77–81)
	Any neoplasm or HPP	4611	184	86 (81–91)	1228	81 (79–83)	1412	82 (80–84)

Main analysis assuming that FS reaches and visualizes descending colon

Strategies in *italic* would not automatically refer subjects with any distal AN to colonoscopy

*CRC* colorectal cancer, *AA* advanced adenoma, *AN* advanced neoplasia, *HPP* hyperplastic polyp

^a^Histology-defined AN: high-grade dysplasia, (tubulo-)villous histology, CRC, or any combination thereof

^b^This number refers to participants in whom all proximal and distal AN are detected. It is smaller than the sum of participants with CRC or AA detected as their most advanced finding in case of no referral, because those detected with distal CRC may still have proximal AA that would not be detected in case of no referral

The SCORE criteria would have performed similarly to the UK criteria. Among both sexes combined, approximately one out of six AN missed by the UK criteria would have been detected using the NORCCAP criteria (sensitivities: 75 and 79%, respectively). Due to the high number of colonoscopies in men, PLCO criteria would have had the highest overall sensitivity for AN (85%) and the by far largest number of colonoscopies (5229 compared to between 1421 and 3220 for the other trials’ criteria). In women, the NORCCAP criteria would have achieved higher sensitivities for AN (73%) than the PLCO criteria (72%), but would also have required more colonoscopies (1216 vs. 1062). The differences in sensitivities for AN between different referral strategies were larger in men than in women. Gender differences in sensitivity within the same strategy were most pronounced in the US (PLCO) criteria (20%-points for any AN), which consider age and sex in addition to colonoscopic findings. In all other strategies, differences were between 7 and 10%-points.

Comparing referral criteria used in FS trials and further conceivable strategies, including the widely recommended criterion “any distal neoplasm” [2–4], a similar range of overall and sex-specific sensitivities and numbers of colonoscopies would be expected. With most criteria, the majority of advanced proximal neoplasms, 54-88%, would still be expected to be missed (Table 4). The US criteria were the only criteria to detect more than half of all proximal ANs (55%). This was driven by the high sensitivity among men (75%), whereas more than three out of four AN would still have been missed in women (sensitivity 23%). Overall, similar patterns emerged when assuming that FS reaches the sigmoid colon only (Supplementary Table 1). For CRC, sensitivities were 1-5%-points lower. For AA and any AN, 3-6%-points lower sensitivities were achievable.

**Table 4:** Numbers and shares of participants referred to colonoscopy and numbers of colonoscopies needed to detect one proximal advanced neoplasm according to different colonoscopy referral strategies after FS

Colonoscopy referral criterion after FS screening	Men (N = 7323, incl. 359 with prox. AN)				Women (N = 7624, incl. 222 with prox. AN)				Total (N = 14,947, incl. 581 with prox. AN)			
	Number of colonoscopies N (%)^a^	Prox. AN detected N (%)^b^	NCN	Δ NCN	Number of colonoscopies N (%)^a^	Prox. AN detected N (%)^b^	NCN	Δ NCN	Number of colonoscopies N (%)^a^	Prox. AN detected N (%)^b^	NCN	Δ NCN
UK FS screening trial	965 (13)	114 (32)	8.5	9.8	456 (6)	29 (13)	15.7	16.9	1421 (10)	143 (25)	9.9	11.5
SCORE	1146 (16)	125 (35)	9.2	10.9	586 (8)	34 (15)	17.2	18.9	1732 (12)	159 (27)	10.9	12.9
NORCCAP	2004 (27)	162 (45)	12.4	15.2	1216 (16)	58 (26)	21.0	22.6	3220 (22)	220 (38)	14.6	17.5
*US (PLCO)*	*4167 (57)*	*270 (75)*	*15.4*	*17.6*	*1062 (14)*	*50 (23)*	*21.2*	*23.3*	*5229 (35)*	*320 (55)*	*16.3*	*18.5*
≥*2 neoplasms, *≥* 1 AN*	*395 (5)*	*56 (16)*	*7.1*	*Ref.*	*152 (2)*	*11 (5)*	*13.8*	*Ref.*	*547 (4)*	*67 (12)*	*8.2*	*Ref.*
≥*2 neoplasms*	*630 (9)*	*74 (21)*	*8.5*	*13.1*	*263 (3)*	*16 (7)*	*16.4*	*22.2*	*893 (6)*	*90 (15)*	*9.9*	*15.0*
*Histology*-*defined AN*^*c*^	*617 (8)*	*75 (21)*	*8.2*	*11.7*	*307 (4)*	*20 (9)*	*15.4*	*17.2*	*924 (6)*	*95 (16)*	*9.7*	*13.5*
*AN *>* 1* *cm*	*639 (9)*	*76 (21)*	*8.4*	*12.2*	*314 (4)*	*16 (7)*	*19.6*	*32.4*	*953 (6)*	*92 (16)*	*10.4*	*16.2*
Any AN	854 (12)	102 (28)	8.4	10.0	422 (6)	27 (12)	15.6	16.9	1276 (9)	129 (22)	9.9	11.8
Any neoplasm	1941 (27)	156 (43)	12.4	15.5	1186 (16)	56 (25)	21.2	23.0	3127 (21)	212 (36)	14.8	17.8
Any neoplasm or HPP	2737 (37)	191 (53)	14.3	17.3	1874 (25)	74 (33)	25.3	27.3	4611 (31)	265 (46)	17.4	20.5

KolosSal study population (N = 14,947), main analysis assuming that FS reaches and visualizes descending colon

^a^% of all screenees

Strategies in *italic* would not automatically refer subjects with any distal AN to colonoscopy

*AN* advanced neoplasia (colorectal cancer or advanced adenoma), FS flexible sigmoidoscopy, *HPP* hyperplastic polyp, *NCN* number of colonoscopies needed per proximal AN detected, *ΔNCN* additional number of colonoscopies needed per additionally detected AN compared to the most restrictive strategy in the hierarchy (≥ 2 neoplasms, thereof ≥ 1AN), *Ref.* reference group

^b^% of participants with proximal AN

^c^Histology-defined AN: high-grade dysplasia, (tubulo-)villous histology, colorectal cancer, or any combination thereof

As a consequence of the relatively low prevalence of proximal AN in the study population (581/14,947 = 3.9%), subjects not referred to colonoscopy were unlikely to have proximal AN, despite the low sensitivities of the strategies for their detection: Even without colonoscopy referral, the negative predictive value (NPV) for AN was approximately 95%, increasing to approximately 97% when using more comprehensive referral criteria. NPVs for the endpoint CRC were very close to 100% for all strategies (Supplementary Table 3). Assuming a more limited reach of FS did not change the results materially (Supplementary Table 4).

### Number of colonoscopies per neoplasm detected

From the numbers of colonoscopy referrals and detected proximal AN with each strategy, we calculated the average NCN to detect one proximal AN. As shown in Table 4, 5–57% of men and 2–25% of women would need to undergo colonoscopy. The UK and SCORE strategies, referring approximately one out of seven men and one out of 13 women to colonoscopy, would detect approximately one out of three proximal ANs in men and one out of seven proximal ANs in women each. Applying the NORCCAP and US criteria would detect nearly one out of two and three out of four proximal ANs in men, respectively, but require colonoscopy follow-up in 27% (NORCCAP) and 57% (US) of all men. While only 16% (NORCCAP) and 14% (US) of women would undergo colonoscopy with these strategies, detection of proximal ANs would also remain very limited (only 26 and 23%, respectively).

The NCNs increase gradually with more extensive referral strategies and are substantially higher in strategies with colonoscopy referral after detection of any neoplasm than in those with referral after detection of advanced neoplasms only. The most restrictive strategy examined assumed colonoscopy referral only due to CRC or at least two detected distal neoplasms, requiring one of them to be advanced. That strategy had the lowest NCN among both, men and women: Only 4% of the study population (N = 547) would undergo colonoscopy and 67/581 = 12% of all proximal AN would be detected. Compared to this strategy, requiring 547/67 = 8.2 colonoscopies to detect one proximal AN, a steep increase in the numbers of colonoscopies required to detect an additional AN was observable for all other strategies. This increase was larger with more extensive referral criteria. For example, taking both sexes together, colonoscopy referral due to any AN required 729 additional colonoscopies (1276-547) and detected 62 additional AN (129-67), resulting in an incremental NCN of 729/62 = 11.8. By comparison, referral due to any distal neoplasm would have required 2580 additional colonoscopies for 145 additionally detected AN. The incremental NCN of 17.8 (2580/145) was thereby considerably higher than for the “any AN” referral strategy.

For all strategies, NCN were substantially lower for men than for women. In men, between 7.1 and 15.4 colonoscopies would be conducted per proximal AN detected, assuming FS to visualize the descending colon, whereas between 13.8 and 25.3 colonoscopies would be conducted per proximal AN detected in women. Assuming FS to reach the sigmoid colon only, all NCNs would be slightly lower among men, whereas both slightly higher and slightly lower NCNs were estimated for women (Supplementary Table 2).

## Discussion

We estimated diagnostic performance of a once-only FS for the detection of advanced colorectal neoplasms by modeling different colonoscopy referral criteria following FS in a German CRC screening population. Without colonoscopy referral, assuming that the descending colon is not reached, FS would detect 62% of AN in men and 59% of AN in women. At the upper end, with FS reaching the entire descending colon and colonoscopy referral following the US (PLCO) criteria, 57% of all men and would undergo colonoscopy, yielding sensitivities of 92% for AN. In women, colonoscopy following any neoplasm or hyperplastic polyp would yield the highest number of colonoscopies (25% of all women) and NCN (25.3) and achieve 76% sensitivity for AN. Compared to referral due to distal AN only (NCN 15.6), the number of colonoscopies to detect one proximal AN would strongly increase, and a large share of this increase in sensitivity could be achieved with much lower colonoscopy referral rates using less extensive referral strategies.

We found major sex differences in sensitivity (higher in men) and the NCN to detect one proximal AN (higher in women) that might suggest considering sex-specific referral strategies. Although sensitivity could be increased among women by using less restrictive referral strategies compared to men, this would further aggravate the gender discrepancies in the NCN. For example, assuming FS to reach and visualize the descending colon, referral of women with any distal neoplasm or HPP would approximately yield the same overall sensitivity for detecting any AN (76%) as referral of men with any distal advanced neoplasm only (77%) (see Table 3). However, such an approach would require 25.3 colonoscopies to detect one additional proximal AN among women, three times the corresponding number among men (8.4, see Table 4). To achieve a better use of colonoscopy resources, quite a contrary approach may make sense: Referring men to colonoscopy after detecting any distal neoplasm, but women only after detecting a distal AN would not only result in quite comparable NCN for both sexes (12.4 and 15.6, respectively, see Table 4), but also lead to a higher number of detected proximal AN (156 + 27 = 183 vs. 102 + 74 = 176), despite an overall much lower number of colonoscopies (1941 + 422 = 2363 vs. 854 + 1874 = 2728) compared to the aforementioned “equal sensitivity” scenario.

Several earlier studies had reported an increased risk of proximal AN in the presence of specific distal findings [20–22], supporting the use of colonoscopy referral strategies based on such findings, but only few studies (one each from Japan, Spain and China) have explicitly compared expected performance of various referral strategies in terms of detection of proximal AN [13–15]. Like our study, they reported increased sensitivity for proximal AN with more extensive referral strategies, and two of them [14, 15] also reported major sex differences. Castells et al. [14] obtained virtually identical sensitivities for proximal AN using the UK, SCORE and NORCCAP criteria (22, 31 and 37%, respectively). Similar to our study, sensitivities were consistently higher in men than in women. The US strategy, where the largest sex-specific differences could be expected, was not examined, though. In the study of Wong et al. [15], estimated achievable sensitivities for proximal AN using the SCORE and NORCCAP strategies were very similar to those obtained in our study, with 31 and 38%, respectively. Compared to our study, they found lower sensitivities when using the UK criteria (14 vs. 25%) or the US criteria (42 vs. 55%). Our study expands the evidence from these previous studies in several important respects, in that we included a much larger number of potential referral strategies (12 compared to 2, 3 or 4), along with a larger number of participants with proximal AN (581 compared to 319, 212, and 132, respectively). Furthermore, we provided, for the first time, detailed sex specific analyses for different assumptions regarding the reach of FS. The mean age of the so far largest study population from Japan (48 years) had been below the typical age at which FS is recommended and conducted, and this study had assessed only two referral strategies (any distal AN versus any distal neoplasm) for both sexes combined [13]. Our study is the first to explicitly quantify the “incremental NCN”, i.e., the number of additional colonoscopies needed to detect one additional proximal AN when comparing more extensive to more restrictive referral strategies. Such “incremental NCN”, which we reported for comparisons of referral strategies to the most restrictive strategy under investigation could be derived analogously for comparisons between any other pairs of more restrictive and more comprehensive referral strategy from the data presented in this paper and may be a parameter of particular relevance for delineating the study population that should be referred.

In contrast to a previous study indicating that histology-defined AAs discovered during FS are stronger predictors for proximal AN than large distal adenomas [21], we did not find pronounced differences in sensitivity when using different definitions of AA for referral strategies. However, referral based on size-defined AN tended to perform worse in terms of NCN and ΔNCN than referral based on histology-defined AN. Compared to other primary colonoscopy screening populations [23–27], the CRC detection rate was somewhat higher in our study (1.4%). Similarly, estimated sensitivities of FS for AN detection were mostly higher than previously estimated from meta-analysis results [28]. Possible reasons for the relatively high CRC detection rate include the substantially older age of the study population compared to other studies, conduction of the study in a high incidence country and a high incidence region within Germany [29], and exclusion of subjects with a previous colonoscopy in the past 5 years who have very low CRC detection rates [17]. Despite stringent in- and exclusion criteria applied in our study, it cannot be ruled out entirely that some subjects who underwent screening colonoscopy had other symptoms that motivated them to visit a gastroenterology practice. However, our study thereby accurately reflects the setting in which subjects are recruited in the German colonoscopy screening program. This self-selection might have further contributed to the somewhat higher CRC prevalence in our study. One consequence are consistently lower NCNs than those found in a previous study [27].

Via adenoma removal during FS, a share of CRCs can actually be prevented. Compared to screening colonoscopy, some proximal neoplasms are inevitably missed, thus limiting the preventive potential of FS-only screening. Results from the UK Flexible Sigmoidoscopy Screening Trial with its comparably restrictive referral criteria indicate that even a single FS can achieve a significant and long-lasting reduction of distal CRC incidence and mortality [8]. Reductions of proximal colon cancer incidence were not seen, suggesting that much higher colonoscopy referral rates would be needed to have a significant effect on proximal colon cancer incidence. Whether expanding the colonoscopy referral rates would be the best way to enhance sensitivity and effectiveness of FS based screening appears questionable in the light of our results. Other approaches, such as conduction of a single FIT first, using a positive FIT result as referral criterion to colonoscopy, followed by a once-only FS in FIT negatives [28, 30] are potentially more promising. In the NORCCAP study, CRC incidence and mortality of FS screening alone were also compared to a combination with FIT [7]. The study found no significant differences in detection rates of adenomas or CRC and statistically non-significant reductions in CRC mortality in both groups. Incidence was higher and mortality lower in the group with combined FIT and FS screening compared to sole FS screening. In a previous approach of modeling performance of FS, alone and combined with FIT [28], no individual patient data was available and thus, performance of FS could not be examined for different referral strategies. Estimated overall sensitivities of FS for AN detection were 60, 68 and 72%, assuming colonoscopy referral rates of 0, 20 and 30%, respectively. In the present study, estimated sensitivities for AN were somewhat higher, with 66, 74 and 79% at comparable referral rates (0, 22 and 36% using no referral, any AN or any neoplasm as referral criteria, respectively). Another recent study [30] investigated performance of a single FS alone and combined with FIT for the widely recommended referral criterion due to any distal neoplasm. With sensitivities of 86% and 72% of FS alone for CRC and AA, respectively, estimated accuracy of FS was similar to that of the present study. Additional conduction of FIT was estimated to increase sensitivities for CRC to 100% and sensitivities for AA to at least 72% and up to 82%, depending on the FIT cutoff.

Our study has several strengths. To our knowledge, it is the most comprehensive analysis of colonoscopy referral criteria, examining 12 different criteria in a very large population of participants of screening colonoscopy. It is the first to explicitly quantify the incremental benefit and burden of extending a relatively restrictive colonoscopy referral strategy following FS. Sensitivities for proximal and for any AN were investigated. All included participants underwent full colonoscopy, ensuring that sensitivities of FS are not overestimated due to missed proximal neoplasms at an incomplete colonoscopy. Participants underwent colonoscopy for primary screening, not for clarification of symptoms. Thus, potential overestimation of sensitivity of FS if symptomatic subjects with a presumably higher prevalence of FS-reachable findings had been included should have been avoided. To our knowledge, our study is the by far largest of its kind, with more than 15,000 participants, including over 1500 AA and 200 CRC cases. For all participants, detailed colonoscopy data were available, including location, size and histopathologic features of every finding. These comprehensive data allowed us to provide precise gender-specific estimates of sensitivity, NCN and the NPV for a range of FS-based screening strategies. Assuming a more limited reach of FS did not alter any of the results materially.

Our study also has limitations. Sigmoidoscopy results were derived from colonoscopy. Although a common approach in studies investigating sensitivity of FS [13–15, 21, 22], performance of FS might differ from that of colonoscopy, e.g. due to different bowel preparation procedures (enema vs. oral bowel cleansing) or the absence of sedation. Evidence in this matter is inconclusive, however [31]. On the other hand, the more convenient preparation procedure of FS might yield higher participation rates which might offset a somewhat smaller sensitivity in the distal colon and rectum compared to colonoscopy. Furthermore, also colonoscopy may miss neoplasms, mainly in the proximal colon. Participants with incomplete colonoscopy (0.9%, N = 164/19,261) were excluded from our analyses. Not reaching the coecum will slightly diminish sensitivity of colonoscopy in screening practice. Another factor potentially influencing sensitivity of FS is conduction of the procedure by non-gastroenterologists. Although FS, in contrast to colonoscopy, is frequently conducted by general practitioners and may even by conducted by nurses [32], detection rates are expected to be similar to those of gastroenterologists [33, 34]. Nevertheless, a certain fraction of FS exams will not be completed in practice, e.g. due to pain or insufficient bowel preparation. Those factors might reduce insertion depth and thereby sensitivity of FS somewhat, although our sensitivity analyses indicated that results were very similar even when only the rectum and sigmoid colon were assumed to be reached and visualized by FS. Full compliance to follow-up colonoscopy due to the examined referral criteria was assumed. Partial non-adherence would reduce the gain in achievable sensitivities compared to FS without colonoscopy referral. For example, assuming only 75% adherence rate to colonoscopy follow-up after recommended referral would reduce the 24 percentage points increase in sensitivity for AN achievable by applying the US criteria to men to approximately 18 percentage points. In some of the investigated strategies, we assumed that distal AN discovered during FS would be removed immediately without colonoscopy follow-up. Although advanced adenomas can in principle be removed during FS, the American Society of Gastrointestinal Endoscopists (ASGE) recommends polypectomy during FS only after adequate bowel preparation [35]. Finally, our analyses did not consider sessile serrated polyps which were not systematically detected and recorded by many endoscopists during the earlier years of study recruitment.

In conclusion, FS applied with a moderately restrictive colonoscopy referral strategy, such as referral only due to advanced distal findings, would likely achieve high sensitivities for detection of ANs in women, and even higher sensitivities in men, in a large German CRC screening population and require considerably fewer colonoscopies than primary screening colonoscopy. Even for moderately restrictive referral strategies, the share of false-negatives, i.e. of missing a proximal AA or even proximal colon cancer if FS is not followed by colonoscopy, was very low in our study. However, for any referral strategy, much higher numbers of colonoscopies to detect one proximal AN would be needed for women than for men, and these numbers would substantially increase with increasingly extensive referral strategies. Restricting colonoscopy referral to those with any advanced distal neoplasm rather than the commonly recommended referral of those with any distal neoplasm, at least among women, should be considered. A drawback of such a strategy would be a potentially higher number of interval cancers. On the other hand, even a single FIT detects the majority of proximal colon cancers and a significant proportion of proximal advanced adenomas [28, 36]. To avoid an increase in the number of interval cancer cases, additional conduction of FIT prior to FS, with colonoscopy referral of FIT-positives and conduction of FS among FIT-negatives might be an alternative.

## Electronic supplementary material

Below is the link to the electronic supplementary material.

Supplementary material 1 (DOCX 92 kb)

## Abbreviations

AA: Advanced adenoma
AN: Advanced neoplasm
CI: Confidence interval
CRC: Colorectal cancer
FS: Flexible sigmoidoscopy
NCN: Number of colonoscopies needed
