# Supplementary material for: Flexible sigmoidoscopy in colorectal cancer screening: implications of different colonoscopy referral strategies
Source: Eur J Epidemiol. 2018 May 12;33(5):473–84. doi: 10.1007/s10654-018-0404-x (PMC5968045; doi:10.1007/s10654-018-0404-x)
Supplement: Supplementary file 1 — Supplementary material 1 (DOCX 92 kb) [file 10654_2018_404_MOESM1_ESM.docx]

**SUPPLEMENT TO:**

**Flexible sigmoidoscopy in colorectal cancer screening:**

**implications of different colonoscopy referral strategies**

Tobias Niedermaier PhD student^1,2^, Korbinian Weigl PhD student^1,2,4^, Michael Hoffmeister senior researcher in epidemiology^1^, Hermann Brenner professor of epidemiology^1,3,4^

Author affiliations:

^1^Division of Clinical Epidemiology and Aging Research, German Cancer Research Center (DKFZ), Heidelberg, Germany

^2^Medical Faculty Heidelberg, University of Heidelberg, Heidelberg, Germany

^3^Division of Preventive Oncology, German Cancer Research Center (DKFZ) and National Center for Tumor Diseases (NCT), Heidelberg, Germany

^4^German Cancer Consortium (DKTK), German Cancer Research Center (DKFZ), Heidelberg, Germany

**Corresponding author:**

Hermann Brenner

Division of Clinical Epidemiology and Aging Research, German Cancer Research Center

Im Neuenheimer Feld 581, D-69120 Heidelberg, Germany

Phone ++49-6221-421300, Fax ++49-6221-421302

E-mail h.brenner@dkfz.de

**Supplementary Table 1.** Expected overall sensitivities in % (95% CIs) of flexible sigmoidoscopy (FS) based screening with different colonoscopy referral strategies in the male and female KolosSal study population, sorted by the number of colonoscopies conducted. Sensitivity analysis assuming that FS reaches and visualizes sigmoid colon.

| **Sex** | Colonoscopy referral criterion after FS screening | Number of colono-scopies | Most advanced finding at colonoscopy | | | | | |
| --- | --- | --- | --- | --- | --- | --- | --- | --- |
|  |  |  | **CRC** (N=140 / 73) | | **AA** (N=971 / 544) | | **Any AN** (N=1,111 / 617) | |
|  |  |  | N  detected | Sensitivity [%]  (95% CI) | N  detected | Sensitivity [%]  (95% CI) | N  detected^b^ | Sensitivity [%]  (95% CI) |
|  | | | | | | | | |
| Men | *No referral* | *0* | *114* | *81 (74-87)* | *585* | *60 (57-63)* | *685* | *62 (59-65)* |
|  |  |  |  |  |  |  |  |  |
|  | UK FS screening trial | 882 | 122 | 87 (80-92) | 684 | 70 (67-73) | 806 | 73 (70-75) |
|  | SCORE | 1,051 | 122 | 87 (80-92) | 694 | 71 (69-74) | 816 | 73 (71-76) |
|  | NORCCAP | 1,769 | 123 | 88 (81-93) | 733 | 75 (73-78) | 856 | 77 (74-79) |
|  | *US (PLCO)* | *4,064* | *134* | *96 (91-98)* | *863* | *89 (87-91)* | *997* | *90 (88-91)* |
|  |  |  |  |  |  |  |  |  |
|  | *≥2 neoplasms, ≥1 AN* | *334* | *117* | *84 (76-89)* | *623* | *64 (61-67)* | *740* | *67 (64-69)* |
|  | *≥2 neoplasms* | *521* | *118* | *84 (77-90)* | *639* | *66 (63-69)* | *757* | *68 (65-71)* |
|  | *Histology-defined AN^a^* | *585* | *120* | *86 (79-91)* | *649* | *67 (64-70)* | *769* | *69 (66-72)* |
|  | *AN >1 cm* | *589* | *118* | *84 (77-90)* | *652* | *67 (64-70)* | *770* | *69 (67-72)* |
|  | Any AN | 796 | 121 | 86 (80-92) | 675 | 70 (67-72) | 796 | 72 (69-75) |
|  | Any neoplasm | 1,710 | 123 | 88 (81-93) | 727 | 75 (72-78) | 850 | 77 (74-79) |
|  | Any neoplasm or HPP | 2,524 | 125 | 89 (83-94) | 767 | 79 (76-82) | 892 | 80 (78-83) |
|  | | | | | | | | |
| Women | *No referral* | *0* | *50* | *68 (57-79)* | *317* | *58 (54-62)* | *365* | *59 (55-63)* |
|  |  |  |  |  |  |  |  |  |
|  | UK FS screening trial | 414 | 50 | 68 (57-79) | 339 | 62 (58-66) | 389 | 63 (59-67) |
|  | SCORE | 534 | 50 | 68 (57-79) | 343 | 63 (59-67) | 393 | 64 (60-67) |
|  | *US (PLCO)* | *964* | *56* | *77 (65-86)* | *359* | *66 (62-70)* | *415* | *67 (63-71)* |
|  | NORCCAP | 1,090 | 56 | 77 (65-86) | 366 | 67 (63-71) | 422 | 68 (65-72) |
|  |  |  |  |  |  |  |  |  |
|  | *≥2 neoplasms, ≥1 AN* | *136* | *50* | *68 (57-79)* | *326* | *60 (56-64)* | *376* | *61 (57-65)* |
|  | *≥2 neoplasms* | *225* | *50* | *68 (57-79)* | *330* | *61 (56-65)* | *380* | *62 (58-65)* |
|  | *Histology-defined AN^a^* | *287* | *50* | *68 (57-79)* | *332* | *61 (57-65)* | *382* | *62 (58-66)* |
|  | *AN >1 cm* | *287* | *50* | *68 (57-79)* | *327* | *60 (56-64)* | *377* | *61 (57-65)* |
|  | Any AN | 387 | 50 | 68 (57-79) | 337 | 62 (58-66) | 387 | 63 (59-67) |
|  | Any neoplasm | 1,060 | 56 | 77 (65-86) | 363 | 67 (63-71) | 419 | 68 (64-72) |
|  | Any neoplasm or HPP | 1,735 | 56 | 77 (65-86) | 384 | 71 (67-74) | 440 | 71 (68-75) |

**Supplementary Table 1, continued**

| **Sex** | Colonoscopy referral criterion after FS screening | Number of colono-scopies | Most advanced finding at colonoscopy | | | | | |
| --- | --- | --- | --- | --- | --- | --- | --- | --- |
|  |  |  | **CRC** (N=213) | | **AA** (N=1,515) | | **Any AN** (N=1,728) | |
|  |  |  | N  detected | Sensitivity [%]  (95% CI) | N  detected | Sensitivity [%]  (95% CI) | N  detected^b^ | Sensitivity [%]  (95% CI) |
|  | | | | | | | | |
| Both sexes | *No referral* | *0* | *164* | *77 (71-82)* | *902* | *59 (57-62)* | *1,050* | *61 (58-63)* |
|  |  |  |  |  |  |  |  |  |
|  | UK FS screening trial | 1,296 | 172 | 81 (75-86) | 1,023 | 68 (65-70) | 1,195 | 69 (67-71) |
|  | SCORE | 1,585 | 172 | 81 (75-86) | 1,037 | 68 (66-71) | 1,209 | 70 (68-72) |
|  | NORCCAP | 2,859 | 179 | 84 (78-89) | 1,099 | 73 (70-75) | 1,278 | 74 (72-76) |
|  | *US (PLCO)* | *5,028* | *190* | *89 (84-93)* | *1,222* | *81 (79-83)* | *1,412* | *82 (80-84)* |
|  |  |  |  |  |  |  |  |  |
|  | *≥2 neoplasms, ≥1 AN* | *470* | *167* | *78 (72-84)* | *949* | *63 (60-65)* | *1,116* | *65 (62-67)* |
|  | *≥2 neoplasms* | *746* | *168* | *79 (73-84)* | *969* | *64 (61-66)* | *1,137* | *66 (64-68)* |
|  | *Histology-defined AN^a^* | *872* | *170* | *80 (74-85)* | *981* | *65 (62-67)* | *1,151* | *66 (64-69)* |
|  | *AN >1 cm* | *876* | *168* | *79 (73-84)* | *979* | *65 (62-67)* | *1,147* | *66 (64-69)* |
|  | Any AN | 1,183 | 171 | 80 (74-85) | 1,012 | 67 (64-69) | 1,183 | 68 (66-71) |
|  | Any neoplasm | 2,770 | 179 | 84 (78-89) | 1,090 | 72 (70-74) | 1,269 | 73 (71-76) |
|  | Any neoplasm or HPP | 4,259 | 181 | 85 (79-89) | 1,151 | 76 (74-78) | 1,332 | 77 (75-79) |

^a^ Histology-defined AN: high-grade dysplasia, (tubulo-)villous histology, CRC, or any combination thereof. Abbreviations: CRC, colorectal cancer; AA, advanced adenoma; AN, advanced neoplasia; HPP, hyperplastic polyp; ^1^24 AAs with missing location information.

^b^ This number refers to participants in whom all proximal and distal AN are detected. It is smaller than the sum of participants with CRC or AA detected as their most advanced finding in case of no referral, because those detected with distal CRC may still have proximal AA that would not be detected in case of no referral.

Strategies in *italic* would not automatically refer subjects with any distal AN to colonoscopy.

**Supplementary Table 2.** Numbers and shares of participants with detected advanced proximal neoplasms and numbers of colonoscopies needed to detect one proximal advanced neoplasm according to different colonoscopy referral strategies after FS. KolosSal study population (N=14,947), sensitivity analysis assuming that FS reaches and visualizes sigmoid colon.

| Colonoscopy referral criterion after FS screening | **Men** (N=7,323,  incl. 426 with prox. AN) | | | | | **Women** (N=7,624,  incl. 252 with prox. AN) | | | | | **Total** (N=14,947,  incl. 678 with prox. AN) | | | | |
| --- | --- | --- | --- | --- | --- | --- | --- | --- | --- | --- | --- | --- | --- | --- | --- |
|  | Number of colonoscopies N (%)^a^ | | Prox. AN detected  N (%)^b^ | NCN | Δ  NCN | Number of colonoscopies N (%)^a^ | | Prox. AN detected  N (%)^b^ | NCN | Δ  NCN | Number of colonoscopies N (%)^a^ | | Prox. AN detected  N (%)^b^ | NCN | Δ  NCN |
|  |  |  |  |  |  |  |  |  |  |  |  |  |  |  |  |
| UK FS screening trial | 882 | (12) | 121 (28) | 7.3 | 8.3 | 414 | (5) | 24 (10) | 17.3 | 21.4 | 1,296 | (9) | 145 (21) | 8.9 | 10.5 |
| SCORE | 1,051 | (14) | 131 (31) | 8.0 | 9.4 | 534 | (7) | 28 (11) | 19.1 | 23.4 | 1,585 | (11) | 159 (23) | 10.0 | 12.0 |
| NORCCAP | 1,769 | (24) | 171 (40) | 10.3 | 12.4 | 1,090 | (14) | 57 (23) | 19.1 | 20.7 | 2,859 | (19) | 228 (34) | 12.5 | 14.7 |
| *US (PLCO)* | *4,064* | *(55)* | *312 (73)* | *13.0* | *14.5* | *964* | *(13)* | *50 (20)* | *19.3* | *21.2* | *5,028* | *(34)* | *362 (54)* | *13.9* | *15.4* |
|  |  |  |  |  |  |  |  |  |  |  |  |  |  |  |  |
| *≥2 neoplasms, ≥1 AN* | *334* | *(5)* | *55 (13)* | *6.1* | *Ref.* | *136* | *(2)* | *11 (4)* | *12.4* | *Ref.* | *470* | *(3)* | *66 (10)* | *7.1* | *Ref.* |
| *≥2 neoplasms* | *521* | *(7)* | *72 (17)* | *7.2* | *11.0* | *225* | *(3)* | *15 (6)* | *15.0* | *22.3* | *746* | *(5)* | *87 (13)* | *8.6* | *13.1* |
| *Histology-defined AN^c^* | *585* | *(8)* | *84 (20)* | *7.0* | *8.7* | *287* | *(4)* | *17 (7)* | *16.9* | *25.2* | *872* | *(6)* | *101 (15)* | *8.6* | *11.5* |
| *AN >1 cm* | *589* | *(8)* | *85 (20)* | *6.9* | *8.5* | *287* | *(4)* | *12 (5)* | *23.9* | *151* | *876* | *(6)* | *97 (14)* | *9.0* | *13.1* |
| Any AN | 796 | (11) | 111 (26) | 7.2 | 8.3 | 387 | (5) | 22 (9) | 17.6 | 22.8 | 1,183 | (8) | 133 (20) | 8.9 | 10.6 |
| Any neoplasm | 1,710 | (23) | 165 (39) | 10.4 | 12.5 | 1,060 | (14) | 54 (21) | 19.6 | 21.5 | 2,770 | (19) | 219 (32) | 12.6 | 15.0 |
| Any neoplasm or HPP | 2,524 | (34) | 207 (49) | 12.2 | 14.4 | 1,735 | (23) | 75 (30) | 23.1 | 25.0 | 4,259 | (28) | 282 (42) | 15.1 | 17.5 |

^a^ % of all screenees

^b^ % of participants with proximal AN

^c^ Histology-defined AN: high-grade dysplasia, (tubulo-)villous histology, CRC, or any combination thereof.

Abbreviations: AN, advanced neoplasia (colorectal cancer or advanced adenoma); FS, flexible sigmoidoscopy; HPP, hyperplastic polyp; NCN, number of colonoscopies needed per proximal AN detected. ΔNCN, additional number of colonoscopies needed per additionally detected AN compared to the most restrictive strategy in the hierarchy (≥2 neoplasms, thereof ≥1AN); Ref. reference group.

Strategies in *italic* would not automatically refer subjects with any distal AN to colonoscopy.

**Supplementary Table 3.** Negative predictive values according to different colonoscopy referral strategies after FS. KolosSal study population (N=14,947), main analysis assuming that FS reaches and visualizes descending colon.

| **Sex** | Colonoscopy referral criterion after FS screening | Number of colono­scopies | Most advanced finding at colonoscopy | | | | | |
| --- | --- | --- | --- | --- | --- | --- | --- | --- |
|  |  |  | **CRC** (N=140 / 73) | | **AA** (N=971 / 544) | | **Any AN** (N=1,111 / 617) | |
|  |  |  | TN / (TN+FN) | NPV [%]  (95% CI) | TN / (TN+FN) | NPV [%]  (95% CI) | TN / (TN+FN) | NPV [%]  (95% CI) |
| Men | *No referral* | *0* | *7,301/7,323* | *99.7 (99.5-99.8)* | *6,858/7,183* | *95.5 (95.0-95.9)* | *6,964/7,323* | *95.1 (94.6-95.6)* |
|  |  |  |  |  |  |  |  |  |
|  | UK FS screening trial | 965 | 6,344/6,358 | 99.8 (99.6-99.9) | 6,113/6,344 | 96.4 (95.9-96.8) | 6,113/6,358 | 96.1 (95.6-96.6) |
|  | SCORE | 1,146 | 6,163/6,177 | 99.8 (99.6-99.9) | 5,943/6,163 | 96.4 (95.9-96.9) | 5,943/6,177 | 96.2 (95.7-96.7) |
|  | NORCCAP | 2,004 | 5,306/5,319 | 99.8 (99.6-99.9) | 5,122/5,306 | 96.5 (96.0-97.0) | 5,122/5,319 | 96.3 (95.8-96.8) |
|  | *US (PLCO)* | *4,167* | *3,151/3,156* | *99.8 (99.6-99.9)* | *3,067/3,151* | *97.3 (96.7-97.9)* | 3,067/3,156 | *97.2 (96.5-97.7)* |
|  |  |  |  |  |  |  |  |  |
|  | *≥2 neoplasms, ≥1 AN* | *395* | *6,910/6,915* | *99.9 (99.8-100)* | *6,625/6,709* | *98.7 (98.5-99.0)* | *6,625/6,714* | *98.7 (98.4-98.9)* |
|  | *≥2 neoplasms* | *630* | *6,676/6,681* | *99.9 (99.8-100)* | *6,408/6,492* | *98.7 (98.4-99.0)* | 6,408/6,497 | *98.6 (98.3-98.9)* |
|  | *Histology-defined AN^a^* | *617* | *6,690/6,706* | *99.8 (99.6-99.9)* | *6,422/6,690* | *96.0 (95.5-96.5)* | *6,422/6,706* | *95.8 (95.3-96.2)* |
|  | *AN >1 cm* | *639* | *6,666/6,684* | *99.7 (99.6-99.8)* | *6,401/6,666* | *96.0 (95.5-96.5)* | *6,401/6,684* | *95.8 (95.3-96.2)* |
|  | Any AN | 854 | 6,454/6,469 | 99.8 (99.6-99.9) | 6,212/6,454 | 96.3 (95.8-96.7) | 6,212/6,469 | 96.0 (95.5-96.5) |
|  | Any neoplasm | 1,941 | 5,369/5,382 | 99.8 (99.6-99.9) | 5,179/5,369 | 96.5 (95.9-96.9) | 5,179/5,382 | 96.2 (95.7-96.7) |
|  | Any neoplasm or HPP | 2,737 | 4,573/4,586 | 99.7 (99.5-99.8) | 4,418/4,573 | 96.6 (96.0-97.1) | 4,418/4,586 | 96.3 (95.8-96.9) |
| Wom­en | *No referral* | *0* | *7,602/7,624* | *99.7 (99.6-99.8)* | *7,352/7,551* | *97.4 (97.0-97.7)* | *7,402/7,624* | *97.1 (96.7-97.5)* |
|  |  |  |  |  |  |  |  |  |
|  | UK FS screening trial | 456 | 7,147/7,168 | 99.7 (99.6-99.8) | 6,975/7,147 | 97.6 (97.2-97.9) | 6,975/7,168 | 97.3 (96.9-97.7) |
|  | SCORE | 586 | 7,017/7,038 | 99.7 (99.5-99.8) | 6,850/7,017 | 97.6 (97.2-98.0) | 6,850/7,038 | 97.3 (96.9-97.7) |
|  | *US (PLCO)* | *1,062* | *6,546/6,562* | *99.8 (99.6-99.9)* | *6,390/6,546* | *97.6 (97.2-98.0)* | 6,390/6,562 | *97.4 (97.0-97.8)* |
|  | NORCCAP | 1,216 | *6,392/6,408* | *99.8 (99.6-99.9)* | 6,244/6,392 | 97.7 (97.3-98.0) | 6,244/6,408 | 97.4 (97.0-97.8) |
|  |  |  |  |  |  |  |  |  |
|  | *≥2 neoplasms, ≥1 AN* | *152* | *7,451/7,467* | *99.8 (99.7-99.9)* | *7,261/7,417* | *97.9 (97.5-98.2)* | *7,261/7,433* | *97.7 (97.3-98.0)* |
|  | *≥2 neoplasms* | *263* | *7,341/7,357* | *99.8 (99.6-99.9)* | *7,155/7,311* | *97.9 (97.5-98.2)* | 7,155/7,357 | *97.3 (96.9-97.6)* |
|  | *Histology-defined AN^a^* | *307* | *7,296/7,317* | *99.7 (99.6-99.8)* | *7,155/7,296* | *97.5 (97.1-97.9)* | *7,115/7,317* | *97.2 (96.8-97.6)* |
|  | *AN >1 cm* | *314* | *7,289/7,310* | *99.7 (99.6-99.8)* | *7,104/7,289* | *97.5 (97.1-97.8)* | *7,104/7,310* | *97.2 (96.8-97.5)* |
|  | Any AN | 422 | 7,181/7,202 | 99.7 (99.6-99.8) | 7,007/7,181 | 97.6 (97.2-97.9) | 7,007/7,202 | 97.3 (96.9-97.7) |
|  | Any neoplasm | 1,186 | 6,422/6,438 | 99.8 (99.6-99.9) | 6,727/6,422 | 97.7 (97.3-98.0) | 6,727/6,438 | 97.4 (97.0-97.8) |
|  | Any neoplasm or HPP | 1,874 | 5,734/5,750 | 99.7 (99.5-99.8) | 5,602/5,734 | 97.7 (97.3-98.1) | 5,602/5,750 | 97.4 (97.0-97.8) |

**Supplementary Table 3, continued (main analysis)**

| **Sex** | Colonoscopy referral criterion after FS screening | Number of colono­scopies | Most advanced finding at colonoscopy | | | | | |
| --- | --- | --- | --- | --- | --- | --- | --- | --- |
|  |  |  | **CRC** (N=213) | | **AA** (N=1,515) | | **Any AN** (N=1,728) | |
|  |  |  | TN / (TN+FN) | NPV [%]  (95% CI) | TN / (TN+FN) | NPV [%]  (95% CI) | TN / (TN+FN) | NPV [%]  (95% CI) |
| Both sexes | *No referral* | *0* | *14,903/14,947* | *99.7 (99.6-99.8)* | *14,210/14,734* | *96.1 (95.8-96.4)* | *14,366/14,947* | *96.1 (95.8-96.4)* |
|  |  |  |  |  |  |  |  |  |
|  | UK FS screening trial | 1,421 | 13,491/13,526 | 99.7 (99.6-99.8) | 13,088/13,491 | 97.0 (96.7-97.3) | 13,088/13,526 | 96.8 (96.4-97.1) |
|  | SCORE | 1,732 | 13,180/13,215 | 99.7 (99.6-99.8) | 12,793/13,180 | 97.1 (96.8-97.3) | 12,793/13,215 | 96.8 (96.5-97.1) |
|  | NORCCAP | 3,220 | 11,698/11,727 | 99.8 (99.6-99.8) | 11,366/11,698 | 97.2 (96.8-97.5) | 11,366/11,727 | 96.9 (96.6-97.2) |
|  | *US (PLCO)* | *5,229* | *9,697/9,718* | *99.8 (99.7-99.9)* | *9,457/9,697* | *97.5 (97.2-97.8)* | 9,457/9,718 | *97.3 (97.0-97.6)* |
|  |  |  |  |  |  |  |  |  |
|  | *≥2 neoplasms, ≥1 AN* | *547* | *14,361/14,382* | *99.9 (99.8-99.9)* | *13,886/14,126* | *98.3 (98.1-98.5)* | *13,886/14,147* | *98.2 (97.9-98.4)* |
|  | *≥2 neoplasms* | *893* | *14,017/14,038* | *99.9 (99.8-99.9)* | *13,563/13,803* | *98.3 (98.0-98.5)* | 13,563/13,824 | *97.7 (97.5-98.0)* |
|  | *Histology-defined AN^a^* | *924* | *13,986/14,023* | *99.7 (99.6-99.8)* | *13,537/13,986* | *96.8 (96.5-97.1)* | *13,537/14,023* | *96.5 (96.2-96.8)* |
|  | *AN >1 cm* | *953* | *13,955/13,994* | *99.7 (99.6-99.8)* | *13,505/13,955* | *96.8 (96.5-97.1)* | *13,505/13,994* | *96.5 (96.2-96.8)* |
|  | Any AN | 1,276 | 13,635/13,671 | 99.7 (99.6-99.8) | 13,219/13,635 | 96.9 (96.6-97.2) | 13,219/13,671 | 96.7 (96.4-97.0) |
|  | Any neoplasm | 3,127 | 11,791/11,820 | 99.8 (99.6-99.8) | 11,451/11,791 | 97.1 (96.8-97.4) | 11,451/11,820 | 96.9 (96.5-97.2) |
|  | Any neoplasm or HPP | 4,611 | 10,307/10,336 | 99.7 (99.6-99.8) | 10,020/10,307 | 97.2 (96.9-97.5) | 10,020/10,336 | 96.9 (96.6-97.3) |

^a^ Histology-defined AN: high-grade dysplasia, (tubulo-)villous histology, CRC, or any combination thereof.

Abbreviations: AA, advanced adenoma; AN, advanced neoplasia (colorectal cancer or advanced adenoma); CRC, colorectal cancer; FS, flexible sigmoidoscopy; HPP, hyperplastic polyp; TN, true negatives (no proximal AN and no colonoscopy referral); FN, false negatives (proximal AN and no colonoscopy referral); NPV, negative predictive value; CI, confidence interval.

Strategies in *italic* would not automatically refer subjects with any distal AN to colonoscopy.

**Supplementary Table 4.** Negative predictive values according to different colonoscopy referral strategies after FS. KolosSal study population (N=14,947), sensitivity analysis assuming that FS reaches and visualizes sigmoid colon.

| **Sex** | Colonoscopy referral criterion after FS screening | Number of colono-scopies | Most advanced finding at colonoscopy | | | | | |
| --- | --- | --- | --- | --- | --- | --- | --- | --- |
|  |  |  | **CRC** (N=140 / 73) | | **AA** (N=971 / 544) | | **Any AN** (N=1,111 / 617) | |
|  |  |  | TN / (TN+FN) | NPV [%]  (95% CI) | TN / (TN+FN) | NPV [%]  (95% CI) | TN / (TN+FN) | NPV [%]  (95% CI) |
| Men | *No referral* | *0* | *7,297/7,323* | *99.6 (99.5-99.8)* | *6,797/7,183* | *94.6 (94.1-95.1)* | *6,897/7,323* | *94.2 (93.6-94.7)* |
|  |  |  |  |  |  |  |  |  |
|  | UK FS screening trial | 965 | 6,423/6,441 | 99.7 (99.6-99.8) | 6,136/6,423 | 95.5 (95.0-96.0) | 6,136/6,441 | 95.3 (94.7-95.8) |
|  | SCORE | 1,146 | 6,254/6,272 | 99.7 (99.5-99.8) | 5,977/6,254 | 95.6 (95.0-96.1) | 5,977/6,272 | 95.3 (94.7-95.8) |
|  | NORCCAP | 2,004 | 5,537/5,554 | 99.7 (99.5-99.8) | 5,299/5,537 | 95.7 (95.1-96.2) | 5,299/5,554 | 95.4 (94.8-95.9) |
|  | *US (PLCO)* | *4,167* | *3,253/3,259* | *99.8 (99.6-99.9)* | *3,145/3,253* | *96.7 (96.0-97.3)* | *3,145/3,259* | *96.5 (95.8-97.1)* |
|  |  |  |  |  |  |  |  |  |
|  | *≥2 neoplasms, ≥1 AN* | *395* | *6,966/6,972* | *99.9 (99.8-100)* | *6,618/6,726* | *98.4 (98.1-98.7)* | *6,618/6,732* | *98.3 (98.0-98.6)* |
|  | *≥2 neoplasms* | *630* | *6,780/6,786* | *99.9 (99.8-100)* | *6,448/6,556* | *98.4 (98.0-98.6)* | *6,448/6,562* | *98.3 (97.9-98.6)* |
|  | *Histology-defined AN^a^* | *617* | *6,718/6,738* | *99.7 (99.5-99.8)* | *6,396/6,718* | *95.2 (94.7-95.7)* | *6,396/6,738* | *94.9 (94.4-95.4)* |
|  | *AN >1 cm* | *639* | *6,712/6,734* | *99.7 (99.5-99.8)* | *6,393/6,712* | *95.2 (94.7-95.7)* | *6,393/6,734* | *94.9 (94.4-95.4)* |
|  | Any AN | 854 | 6,508/6,527 | 99.7 (99.5-99.8) | 6,212/6,508 | 95.5 (94.9-95.9) | 6,212/6,527 | 95.2 (94.6-95.7) |
|  | Any neoplasm | 1,941 | 5,596/5,613 | 99.7 (99.5-99.8) | 5,352/5,596 | 95.6 (95.1-96.2) | 5,352/5,613 | 95.4 (94.8-95.9) |
|  | Any neoplasm or HPP | 2,737 | 4,784/4,799 | 99.7 (99.5-99.8) | 4,580/4,784 | 95.7 (95.1-96.3) | 4,580/4,799 | 95.4 (94.8-96.0) |
| Wom­en | *No referral* | *0* | *7,601/7,624* | *99.7 (99.5-99.8)* | *7,324/7,551* | *97.0 (96.6-97.4)* | *7,372/7,624* | *96.7 (96.3-97.1)* |
|  |  |  |  |  |  |  |  |  |
|  | UK FS screening trial | 456 | 7,187/7,210 | 99.7 (99.5-99.8) | 6,982/7,187 | 97.1 (96.7-97.5) | 6,982/7,210 | 96.8 (96.4-97.2) |
|  | SCORE | 586 | 7,067/7,090 | 99.7 (99.5-99.8) | 6,866/7,067 | 97.2 (96.7-97.5) | 6,866/7,090 | 96.8 (96.4-97.2) |
|  | *US (PLCO)* | *1,062* | *6,643/6,660* | *99.7 (99.6-99.9)* | *6,458/6,643* | *97.2 (96.8-97.6)* | *6,458/6,660* | *97.0 (96.6-97.4)* |
|  | NORCCAP | 1,216 | 6,517/6,534 | 99.7 (99.6-99.8) | *6,339/6,517* | *97.3 (96.8-97.7)* | *6,339/6,534* | 97.0 (96.5-97.4) |
|  |  |  |  |  |  |  |  |  |
|  | *≥2 neoplasms, ≥1 AN* | *152* | *7,465/7,482* | *99.8 (99.6-99.9)* | *7,247/7,432* | *97.5 (97.1-97.9)* | *7,247/7,449* | *97.3 (96.9-97.6)* |
|  | *≥2 neoplasms* | *263* | *7,376/7,393* | *99.8 (99.6-99.9)* | *7,162/7,347* | *97.5 (97.1-97.8)* | *7,162/7,364* | *97.3 (96.9-97.6)* |
|  | *Histology-defined AN^a^* | *307* | *7,314/7,337* | *99.7 (99.5-99.8)* | *7,102/7,314* | *97.1 (96.7-97.5)* | *7,102/7,337* | *96.8 (96.4-97.2)* |
|  | *AN >1 cm* | *314* | *7,314/7,337* | *99.7 (99.5-99.8)* | *7,097/7,314* | *97.0 (96.6-97.4)* | *7,097/7,337* | *96.7 (96.3-97.1)* |
|  | Any AN | 422 | 7,214/7,237 | 99.7 (99.5-99.8) | 7,007/7,214 | 97.1 (96.7-97.5) | 7,007/7,237 | 96.8 (96.4-97.2) |
|  | Any neoplasm | 1,186 | 6,547/6,564 | 99.7 (99.6-99.8) | 6,366/6,547 | 97.2 (96.8-97.6) | 6,366/6,564 | 97.0 (96.5-97.4) |
|  | Any neoplasm or HPP | 1,874 | 5,872/5,889 | 99.7 (99.5-99.8) | 5,712/5,872 | 97.3 (96.8-97.7) | 5,712/5,889 | 97.0 (96.5-97.4) |

**Supplementary Table 4, continued (sensitivity analysis)**

| **Sex** | Colonoscopy referral criterion after FS screening | Number of colono­scopies | Most advanced finding at colonoscopy | | | | | |
| --- | --- | --- | --- | --- | --- | --- | --- | --- |
|  |  |  | **CRC** (N=140 / 73) | | **AA** (N=971 / 544) | | **Any AN** (N=1,111 / 617) | |
|  |  |  | TN / (TN+FN) | NPV [%]  (95% CI) | TN / (TN+FN) | NPV [%]  (95% CI) | TN / (TN+FN) | NPV [%]  (95% CI) |
| Both sexes | *No referral* | *0* | *14,898/14,947* | *99.7 (99.6-99.8)* | *14,121/14,734* | *95.8 (95.5-96.2)* | *14,269/14,947* | *95.5 (95.1-95.8)* |
|  |  |  |  |  |  |  |  |  |
|  | UK FS screening trial | 1,421 | 13,610/13,651 | 99.7 (99.6-99.8) | 13,118/13,610 | 96.4 (96.1-96.7) | 13,118/13,651 | 96.1 (95.8-96.4) |
|  | SCORE | 1,732 | 13,321/13,362 | 99.7 (99.6-99.8) | 12,843/13,321 | 96.4 (96.1-96.7) | 12,843/13,362 | 96.1 (95.8-96.4) |
|  | NORCCAP | 3,220 | 12,054/12,088 | 99.7 (99.6-99.8) | 11,638/12,054 | 96.5 (96.2-96.9) | 11,638/12,088 | 96.3 (95.9-96.6) |
|  | *US (PLCO)* | *5,229* | *9,896/9,919* | *99.8 (99.7-99.9)* | *9,603/9,896* | *97.0 (96.7-97.4)* | *9,603/9,919* | *96.8 (96.4-97.2)* |
|  |  |  |  |  |  |  |  |  |
|  | *≥2 neoplasms, ≥1 AN* | *547* | *14,431/14,454* | *99.8 (99.8-99.9)* | *13,865/14,158* | *97.9 (97.7-98.2)* | *13,865/14,181* | *97.8 (97.5-98.0)* |
|  | *≥2 neoplasms* | *893* | *14,179/14,156* | *99.8 (99.8-99.9)* | *13,610/13,903* | *97.9 (97.6-98.1)* | *13,610/13,926* | *97.7 (97.5-98.0)* |
|  | *Histology-defined AN^a^* | *924* | *14,032/14,075* | *99.7 (99.6-99.8)* | *13,498/14,032* | *96.2 (95.9-96.5)* | *13,498/14,075* | *95.9 (95.6-96.2)* |
|  | *AN >1 cm* | *953* | *14,026/14,071* | *99.7 (99.6-99.8)* | *13,490/14,026* | *96.2 (95.8-96.5)* | *13,490/14,071* | *95.9 (95.5-96.2)* |
|  | Any AN | 1,276 | 13,722/13,764 | 99.7 (99.6-99.8) | 13,219/13,722 | 96.3 (96.0-96.6) | 13,219/13,764 | 96.0 (95.7-96.4) |
|  | Any neoplasm | 3,127 | 12,143/12,177 | 99.7 (99.6-99.8) | 11,718/12,143 | 96.5 (96.2-96.8) | 11,718/12,177 | 96.2 (95.9-96.6) |
|  | Any neoplasm or HPP | 4,611 | 10,656/10,688 | 99.7 (99.6-99.8) | 10,292/10,656 | 96.6 (96.2-96.9) | 10,292/10,688 | 96.3 (95.9-96.6) |

^a^ Histology-defined AN: high-grade dysplasia, (tubulo-)villous histology, CRC, or any combination thereof. Abbreviations: AN, advanced neoplasia (colorectal cancer or advanced adenoma); FS, flexible sigmoidoscopy; HPP, hyperplastic polyp; TN, true negatives; FN, false negatives; NPV, negative predictive value; CI, confidence interval.

Strategies in *italic* would not automatically refer subjects with any distal AN to colonoscopy.
